# Genetic Deletion of ACE2 Induces Vascular Dysfunction in C57BL/6 Mice: Role of Nitric Oxide Imbalance and Oxidative Stress

**DOI:** 10.1371/journal.pone.0150255

**Published:** 2016-04-12

**Authors:** Luiza A. Rabelo, Mihail Todiras, Valéria Nunes-Souza, Fatimunnisa Qadri, István András Szijártó, Maik Gollasch, Josef M. Penninger, Michael Bader, Robson A. Santos, Natalia Alenina

**Affiliations:** 1 Max-Delbrück-Center for Molecular Medicine, Berlin, Germany; 2 Laboratório de Reatividade Cardiovascular, Universidade Federal de Alagoas, Maceió, Alagoas, Brazil; 3 Instituto Nacional de Ciência e Tecnologia em NanoBiofarmacêutica (NanoBIOFAR); 4 Charité University Medicine, Berlin, Germany; 5 Nephrology/Intensive Care, Charité Campus Virchow Klinikum, Experimental and Clinical Research Center (ECRC), Berlin, Germany; 6 Institute of Molecular Biotechnology of the Austrian Academy of Sciences, Vienna, Austria; 7 Federal University of Minas Gerais, Belo Horizonte, Minas Gerais, Brazil; Maastricht University, NETHERLANDS

## Abstract

Accumulating evidence indicates that angiotensin-converting enzyme 2 (ACE2) plays a critical role in cardiovascular homeostasis, and its altered expression is associated with major cardiac and vascular disorders. The aim of this study was to evaluate the regulation of vascular function and assess the vascular redox balance in ACE2-deficient (ACE2^-/y^) animals. Experiments were performed in 20–22 week-old C57BL/6 and ACE2^-/y^ male mice. Evaluation of endothelium-dependent and -independent relaxation revealed an impairment of *in vitro* and *in vivo* vascular function in ACE2^-/y^ mice. Drastic reduction in eNOS expression at both protein and mRNA levels, and a decrease in ^•^NO concentrations were observed in aortas of ACE2^-/y^ mice in comparison to controls. Consistently, these mice presented a lower plasma and urine nitrite concentration, confirming reduced ^•^NO availability in ACE2-deficient animals. Lipid peroxidation was significantly increased and superoxide dismutase activity was decreased in aorta homogenates of ACE2^-/y^ mice, indicating impaired antioxidant capacity. Taken together, our data indicate, that ACE2 regulates vascular function by modulating nitric oxide release and oxidative stress. In conclusion, we elucidate mechanisms by which ACE2 is involved in the maintenance of vascular homeostasis. Furthermore, these findings provide insights into the role of the renin-angiotensin system in both vascular and systemic redox balance.

## Introduction

The prevalence of cardiovascular diseases is one of the major public health problems [1]. The interplay in the triad renin-angiotensin system (RAS), endothelial function (EF), and oxidative stress plays a key role in cardiovascular and metabolic homeostasis and, thus, in the pathogenesis of these diseases [2,3].

Angiotensin-Converting Enzyme 2 (ACE2), a zinc metalloprotease, is an important component of the RAS [4]. It catalyzes the conversion of angiotensin II (Ang II) to angiotensin-(1–7) (Ang-(1–7)). Therefore, it determines the balance between the vasoconstrictor/pro-oxidative peptide Ang II, which is originally produced by ACE and acts via its receptor AT1, and the vasodilatory/antioxidative peptide Ang-(1–7), which interacts with its receptor Mas [5,6]. Consequently, the ACE2/Ang-(1–7)/Mas axis represents a protective arm of the RAS, which counteracts deleterious actions of the ACE/Ang II/AT1 axis. Accordingly, altered expression and/or activity of ACE2 have been associated with major cardiac and vascular pathophysiologies. ACE2-deficient mice exhibit a higher susceptibility to atherosclerosis [7–9], diabetic nephropathy [10,11], myocardial infarction [12], and cardiac hypertrophy [13–16].

The vascular endothelium is a dynamic and pleiotropic tissue, which synthesizes and releases autacoids. The maintenance of EF is crucial for cardiovascular homeostasis. The release of nitric oxide (^•^NO) plays a pivotal role in EF, mediating the crosstalk between endothelium and smooth muscle cells. Oxidation of ^•^NO by superoxide anion (^•^O_2_^-^) and consequent formation of peroxynitrite (^-^ONOO) is discussed to be an important factor contributing to the development of endothelial dysfunction [2]. Furthermore, one of the pivotal hallmarks of cardiovascular and metabolic diseases, including hypertension, diabetes, atherosclerosis, stroke, and heart failure, is reduction-oxidation (redox) imbalance, a condition, known as oxidative stress [17].

Numerous studies have shown that oxidative stress plays an important role in the development of endothelial dysfunction [2,3,5]. We have previously shown that Mas-deficiency results in an imbalance between ^•^NO and reactive oxygen species (ROS) [18,19]. This oxidative stress contributes to both endothelial dysfunction and elevated blood pressure observed in Mas*-*deficient mice on two different genetic backgrounds, C57BL/6 and FVB/N [18,19]. In line with these observations, increased endothelium-dependent relaxation in response to acetylcholine (ACh) *in vitro* and improved EF *in vivo* has been observed in a transgenic rat model, overexpressing human ACE2 in vascular smooth muscle cells (VSMC) [20].

Collectively, these data suggest that the ACE2/Ang-(1–7)/Mas axis plays an important role in maintaining EF and vascular homeostasis. Whereas the adverse cardiovascular effects in ACE2-deficient mice have been partly explained by an impaired EF and an oxidative/nitrosative imbalance [7,21,22], the molecular mechanisms linking ACE2, ROS and ^•^NO metabolism remain poorly understood. The aim of the present study was to evaluate the effect of genetic deletion of ACE2 on *in vivo* vascular function and on ROS and ^•^NO generating and degrading factors in C57BL/6 mice.

## Materials and Methods

### Animals and treatment protocols

The animal procedures were performed conforming the guidelines from the Directive 2010/63/EU of the European Parliament on the protection of animals used for scientific purposes and the experiments performed for this manuscript were approved (study number G0066/04) by the *Landesamt für Gesundheit und Soziales* (LAGeSo), Berlin. Euthanasia was performed using an overdose of inhalable anesthetics. Mice were maintained in individually ventilated cages (IVC), 34×19×13 cm (Tecniplast^®^ S.p.A., Hohenpeissenberg, Germany) under a 12 hr light/dark cycle, with lights on at 6 am, with free access to standard chow (0.25% sodium, SSNIFF^®^ Spezialitäten, Soest, Germany) and drinking water *ad libitum*. The colony of ACE2-deficient mice on C57BL/6 genetic background [4] was maintained by breeding homozygous ACE2-deficient females (ACE2^-/-^) with hemizygous males (ACE2^-/y^). Independent cohorts of 20–22 week-old ACE2^-/y^ and age matched C57BL/6 control (WT) male mice were used for the following experiments: evaluation of *in vivo* endothelial function; metabolic cages, including urine collection, blood sampling, and organ collections for the biochemical analysis. Basic blood pressure and heart rate were measured by radiotelemetry as described before [19].

### Evaluation of endothelial function in conscious mice

EF was measured *in vivo* as presented elsewhere [19,20]. Briefly, the animals were anesthetized using isoflurane as described above and catheters were implanted using aseptic technique. After control hemodynamic measurements (PowerLab^®^, ADI Instruments Co., Colorado Springs, CO, USA), the endothelium-dependent response was tested by administration of increasing doses of acetylcholine (ACh, Sigma-Aldrich^®^, Seelze, Germany) (25/ 50/ 100/ 200 ng.kg^-1^), followed by a single dose of sodium nitroprusside (SNP, Sigma-Aldrich^®^, Seelze, Germany) (10 μg.kg^-1^) in conscious mice. EF was calculated by the normalization of the ACh response by the SNP response according to the formula: EF = ∆MAP (ACh).∆MAP (SNP)^-1^ [19,20].

### Euthanasia and organ collection

To collect the urine, mice were individually housed in metabolic cages (Tecniplast^®^ S.p.A., Hohenpeissenberg, Germany) for up to 72h with food and water available *ad libitum*. After 48 hours of acclimatization, 24h urine was collected, volume was measured gravimetrically, and the samples were stored at -80°C until further analysis.

Blood samples were collected under ketamine/xylazine anesthesia (100 mg.kg^-1^ ketamine, 10 mg.kg^-1^ xylazine, ip) via cardiac puncture from the right ventricle. Blood was aspirated directly into syringes rinsed with 0.2 M Ethylene Diamine Tetraacetic Acid (EDTA), transferred into prechilled EDTA-coated tubes (Fischer Scientif^®^, Schwerte, Germany) and centrifuged (4,000 rpm) at 4°C for 10 minutes. Plasma was transferred to the new tube and kept at -80°C until the analysis. Mice were dissected immediately after the blood draw. Collected tissues were snap-frozen in liquid nitrogen and stored at -80°C until analysis of protein expression and oxidative/nitrosative-redox balance.

### Isometric contractions of mouse vessels

Male wild-type C57BL/6 and ACE2^-/y^ mice (25–30 g, 8–12 weeks) were killed under isoflurane. The thoracic aortas were removed, quickly transferred to cold (4°C), oxygenated (95% O_2_ / 5% CO_2_) PSS (in mmol.L^-1^: 119 NaCl, 4.7 KCl, 1.2 KH_2_PO_4_, 25 NaHCO_3_, 1.2 Mg_2_SO_4_, 11.1 glucose, and 1.6 CaCl_2_) and dissected into 2 mm rings whereby perivascular fat and connective tissue were removed. Each ring was positioned between two stainless steel wires (diameter 0.0394 mm) in a 5 mL organ bath of a Mulvany Small Vessel Myograph (DMT 610 M; Danish Myo Technology, Denmark). The organ bath was filled with PSS. The bath solution was continuously oxygenated with a gas mixture of 95% O_2_ and 5% CO_2_, and kept at 37°C (pH 7.4). The rings were placed under tension of 0.3 g [23]. The software Chart5 (AD Instruments Ltd. Spechbach, Germany) was used for data acquisition and display. The rings were precontracted with 60 mmol.L^-1^ KCl and equilibrated until a stable resting tension was acquired. Vessels were pre-constricted with 1 μmol.L^-1^ phenylephrine, followed by the determination of ACh- or SNP-induced relaxations (3–3000 nmol.L^-1^). Drugs were added to the bath solution if not indicated otherwise. The magnitude of relaxation caused by ACh or SNP was expressed as the percentage of the isometric contraction evoked by phenylephrine, which was taken as 100%.

### Measurement of plasma nitrite levels, total nitrate and nitrite urine excretion rate

Total nitrate and nitrite urinary excretion rate (NOx) was determined using a commercially available kit (Cayman Chemical^®^, Ann Arbor, MI, USA) and normalized to creatinine concentration (mg.dL^-1^) (Labtest^®^, Belo Horizonte, Brazil). The levels of nitrite in plasma were measured as described previously (Xu et al., 2008).

### Measurement of nitric oxide anion (^•^NO) release produced by aortic segments in response to ACh

Mice were euthanized with a mixture of ketamine/xylazine and thoracic aortas were rapidly removed and cut into ring segments ~2.5–3 mm long, with care taken not to injure the endothelium. To estimate vascular ^•^NO production, we used diaminofluorescein (DAF-FM) enhanced fluorescence (excitation 495 nm and emission 515 nm; TECAN^®^ Infinite 200 PRO plate reader, Männedorf, Switzerland) in the darkness. Briefly, each aortic segment was placed in an individual well of cell culture dishes (24-well) containing 2.0 mL of Krebs-HEPES buffer (in mmol.L^-1^: NaCl 99; KCl 4.7; MgSO_4_ 1.2; KH_2_PO_4_ 1.0; CaCl_2_ 1.9; NaHCO_3_ 25; glucose 11.1, NaHEPES 20; pH 7.4) supplemented with 2 mmol.L^-1^ L-arginine, in a light-protected humidified chamber at 37°C for 30 minutes. Following the initial equilibration, basal fluorescence was recorded for 20 minutes. Subsequently, the aortas were reincubated in the same buffer supplemented with the 5 μmol.L^-1^ DAF-FM (Sigma-Aldrich^®^, Seelze, Germany) for 45 minutes. The measurement of ^•^NO release in response to ACh (10 μmol.L^-1^) was performed on 2 separate rings for 3 minutes. To ensure the specificity of the method the assay was also performed in the presence of L-NAME (100 μmol.L^-1^, Sigma-Aldrich^®^, Seelze, Germany). The amount of released ^•^NO was expressed as arbitrary units per mg of aortic protein [24].

### Evaluation of aortic protein expression by Western blot

After collection and removal of adipose and connective tissue, aortas were pulverized in liquid nitrogen. The pulverized samples were suspended in lysis buffer (pH 7.5; Cell Signaling Technology^®^, Beverly, MA) containing 50 mmol.L^-1^ Tris-HCl, 0.1 mmol.L^-1^ EDTA, 0.1 mmol.L^-1^ EGTA, 0.1% SDS, 0.1% deoxycholate, 1% IGEPAL, and a 1000-fold dilution of a mammalian protease and phosphatase inhibitor cocktail (Roche^®^, Mannheim, Germany). The suspension was centrifuged at 14,000 rpm for 20 min. Total protein levels were determined by the Bradford assay [24]. Equal amounts of protein were loaded and transferred to polyvinyl difluoride membranes. Western blots were performed using commercially available antibodies for eNOS, phosphorylated Ser1177-eNOS, HSP90, AKT, phosphorylated AKT, and β-actin (Dilution: 1:1000, 1:1000, 1:500, 1:1000, 1:500, and 1:1000, respectively) (Cell Signaling Technology^®^, Beverly, MA). After binding of corresponding fluorescent secondary antibodies (Li-COR^®^ Biosciences, Lincoln, USA), protein levels were detected and analyzed by Odyssey^®^ IR scanner (Li-COR^®^ Biosciences, Lincoln, USA).

### Real-time quantitative PCR

Total RNA was extracted from aorta (TRIzol^®^ Reagent, Germany) and 1 μg was reverse- transcribed using M-MLV (Invitrogen^®^, Paisley, United Kingdom) to cDNA. The reaction product was amplified by real time quantitative PCR on the 7900HT Fast System using the GoTaq qPCR Master Mix (Promega^®^, Mannheim, Germany). For all mRNA quantification GAPDH was used as an internal reference. The expression levels were obtained from the cycle threshold (Ct) associated with the exponential growth of the PCR products. Quantitative values for mRNA expression were obtained by the parameter 2 ^−ΔCt^, in which ΔCt represented the subtraction of the GAPDH Ct values from the others. Specific fragments were amplified using following primers. eNOS: forward, 5´ CGT CCT GCA AAC CGT GCA GA; reverse, 5´TCC TGG GTG CGC AAT GTG AG. SOD1: forward, 5’ GAC GGT GTG GCC AAT GTG TC; reverse, 5’ CAA GCG GCT CCC AGC ATT TC. SOD2: forward, 5’ GCG GTC GTG TAA ACC TCA ATA ATG; reverse, 5’ CCA GAG CCT CGT GGT ACT TCT C. UCP-2: forward, 5´GCA TTG GCC TCT ACG ACT CT; reverse, 5´GCT CTG GTA TCT CCG ACC AC. GAPDH: forward, 5´ CCA TCA CCA TCT TCC AGG AG; reverse, 5´GTG GTT CAC ACC CAT CAC AA.

### Arginase activity

Arginase activity was determined by quantitating urea production using a spectrophotometric method as described previously [25]. In brief, 50 μL of filtered aorta homogenate were added to 75 μL of Tris-HCl (50 mmol plus 10 mmol MnCl_2_; pH 7.5) into a 2-mL reaction tube. The contents were then mixed and heated at 55°C for 10 min for enzymatic activation. After this step, components of the reaction were incubated with 50 μL of the substrate L-arginine (0.5 M, pH 9.7) at 37°C for 60 min. To stop the reaction, 400 μL of an acid solution (H_2_SO_4_-H_3_PO_4_-H_2_0 = 1:3:7) were added to the tube. Finally, for the development of the chromogen, 25 μL of α-isonitrosopropiophenone (9% in ethanol) were added, and the mixture was heated at 100°C for 45 min. The urea levels were determined by measuring absorbance at 550 nm (TECAN^®^ Infinite 200 PRO plate reader, Männedorf, Switzerland) and normalized by protein concentration [24]. The data were expressed as nmol.L^-1^ urea per mg protein.

### *In situ* detection of vascular ^•^O_2_^−^ release

Superoxide anion (^•^O_2_^-^) production in cryosections of mouse aorta was detected using the fluorescent probe dihydroethidium (DHE) in a procedure described previously with slight modifications [26,27]. Shortly, fresh segments of upper descending thoracic aorta were frozen in Tissue-Tech^®^ OCT compound, cryosectionned into 7 μm thick sections, mounted on a glass slide, and stored at -80°C until analysis. For the experiments, the tissue sections were incubated with Krebs-HEPES buffer for 30 minutes at 37°C with or without 500 U.mL^-1^ polyethylene glycol-conjugated superoxide dismutase (PEG-SOD; Sigma-Aldrich^®^, Seelze, Germany). Then, fresh buffer containing 2 μmol.L^-1^ DHE (Molecular Probes^TM^, Invitrogen^®^, Paisley, United Kingdom) was applied topically onto each tissue section and incubated for another 30 minutes in a light-protected humidified chamber at 37°C. Images were obtained using a LEICA DM-2500 laser scanning confocal microscope equipped with a krypton/argon laser using identical acquisition settings. For each aortic ring, mean fluorescence was calculated from 3 separate high-power fields. Mean data for the quantification of fluorescence were expressed as intensity per mm^2^

### Lipid peroxidation

Lipid peroxidation was determined by measuring the ThioBarbituric Acid-Reactive Substances (TBARS), as a marker of systemic oxidative stress assayed by malondialdehyde (MDA), in aorta homogenate, plasma, and urine, as described by Ohkawa et al. [28]. In parallel, MDA standards were diluted in the range of 0–4 *μ*mol.L^-1^. TBARS values were expressed in nmol.mL^-1^ MDA equivalents. Absorbance was read at 532 nm (TECAN^®^ Infinite 200 PRO plate reader, Männedorf, Switzerland) and normalized by protein concentration [24].

### Aortic concentrations of hydrogen peroxide

Hydrogen peroxide (H_2_O_2_) concentrations in aorta homogenates were determined by fluorescence in black microplates (Nunclon^®^ Surface, Thermo Fisher Scientific^®^, Vantaa, Finland), using the Amplex^®^ UltraRed Hydrogen Peroxide (10-acetyl-3,7-dihydroxiphenoxazine) Assay (Invitrogen^®^, Paisley, United Kingdom). This reagent, in the presence of peroxidase (horseredish peroxidase, HRP), stoichiometrically reacts with H_2_O_2_ to form a red-fluorescent oxidation product, resorufin. A standard curve of H_2_O_2_ was prepared by dilution into reaction buffer, with concentrations ranging from 0 to 10 μmol.L^-1^. Next, 50μL from the prepared curve points or from the aorta homogenate samples (or plasma) were plated in duplicate, with the addition of 50μL of working solution/HRP for beginning the reaction. The microplates were incubated at room temperature for 120 minutes, protected from light, and read at wave lengths of 530 nm and 590 nm for excitability and emission, respectively, in a microplate reader (Tecan 200 Infinite^®^ 200 PRO plate reader, Männedorf, Switzerland).

### Vascular enzymatic antioxidant status

Superoxide dismutase (SOD) and glutathione peroxidase (GPx) activity in aorta homogenates were measured with specific assay kits (Fluka^®^ and Cayman Chemical^®^, Seelze, Germany, and Ann Arbor, MI, USA, respectively) according to the protocols provided by the manufacturers. Catalase activity in aorta homogenate was measured spectrophotometrically as described elsewhere [19]. All data were normalized by protein concentration [24].

### Data Analysis

Results are expressed as mean ± SEM, and "n" indicates the number of animals used in the experiment. The dose-response curves of the different groups were compared by two way ANOVA followed by *Bonferroni’s* correction. For simple comparisons between 2 groups, an unpaired Student’s *t* test was used where appropriate using GraphPad Prism^®^ version 5.0 for Windows. A value of *P*<0.05 was considered significant.

## Results

### ACE2-deficient mice exhibit *in vivo* vascular dysfunction

**Fig 1:**
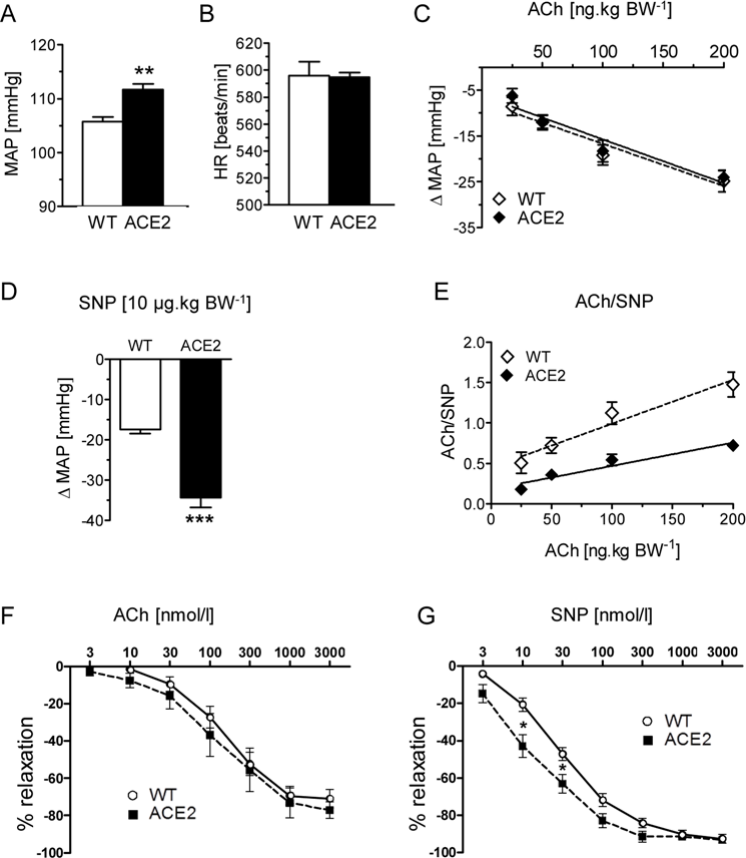
Vascular function in ACE2^-/y^ mice. Mean arterial pressure (**A**) and heart rate (**B**) were measured by radiotelemetry in ACE2^-/y^ and WT (n = 6) mice. Data represent means±SEMs of 3 days of recording. **P<0.01. Vascular response to increasing concentrations [50–200 μg.kg^-1^] of ACh (**C**) and 10 μg.kg^-1^ SNP (**D**) in ACE2^-/y^ (n = 5) and WT (n = 6) mice. (**E**) Vascular response to ACh normalized by SNP. Data represent means±SEMs. **P<0.01 (Student *t* test); §§ *P*<0.01 (2-way ANOVA). Similar data were obtained in 2 independent experiments. (**F**) ACh and (**G**) SNP dose-response curve of aortic rings from ACE2^-/y^ (KO, n = 8–10) and WT (n = 13) mice. Rings were precontracted with phenylephrine (1 μmol.L^-1^) and then stepwise relaxed with ACh or SNP (3–3000 nmol.L^-1^). *P<0.05.

To clarify the physiological role of ACE2 in blood pressure regulation, we first performed radiotelemetric recordings in conscious ACE2^-/y^ male mice. Mean arterial pressure was modestly (~7 mmHg), but significantly elevated in ACE2^-/y^ mice in comparison to WT mice (Fig 1A). In contrast, heart rate was not affected by ACE2 deletion (Fig 1B).

To evaluate if ACE2-deficiency affects vascular function, we investigated the vascular reactivity *in vivo* in conscious ACE2^-/y^ mice by bolus intra-aortic administration of the endothelium-dependent vasodilator acetylcholine (ACh). This mode of ACh application does not reduce the heart rate of the animals (S1 Fig), since ACh is degraded before reaching cardiac rhythm generators, and has been used before by our group to evaluate endothelial function *in vivo* in mice [19] and rats [20]. The vasodilatory response to ACh did not differ between ACE2^-/y^ and WT mice over the dose range of 50 to 200 ng.kg^-1^ (Fig 1C). In contrast, the endothelium-independent response, elicited by SNP, was drastically increased in ACE2^-/y^ mice (Fig 1D), arguing for an adaptation mechanism in the vascular smooth muscle cells. Normalization of the ACh response to the SNP revealed impairment in endothelium-dependent vascular reactivity in ACE2^-/y^ mice (Fig 1E), indicating that genetic deletion of ACE2 may result in a novel equilibrium with an impaired endothelial release of vasodilators and an increased smooth muscle response to these substances.

We could also confirm these data *in vitro* using aortic rings of ACE2^-/y^ and WT mice. While ACh-induced relaxation was unaffected in the ACE2-deficient vessels, SNP elicited a more potent effect at lower doses in ACE2^-/y^ aortas (Fig 1F and 1G).

### ACE2-deficiency leads to reduced ^•^NO bioavailability

**Fig 2:**
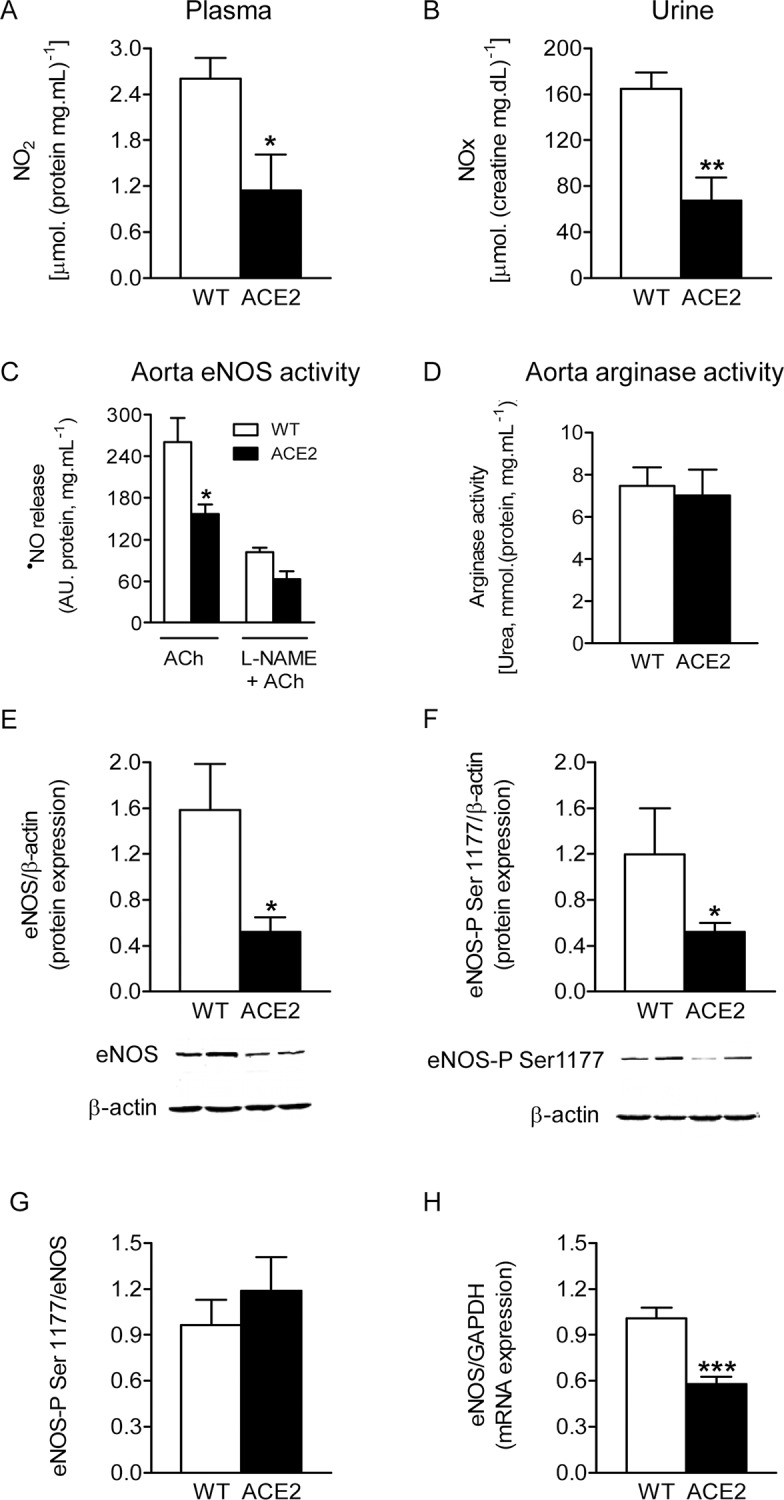
Nitric oxide availability and eNOS expression in ACE2^-/y^ mice. Plasma nitrite content **(A)** and 24-hour urinary excretion (**B**) of nitrate plus nitrite (NOx) in ACE2^-/y^ (n = 6) and WT (n = 6) mice. (**C)** eNOS activity, evaluated by ^•^NO release in aorta rings upon application of ACh (DAF-FM bioassay) (ACE2 n = 5; WT n = 6). (**D**) arginase activity in aorta homogenates of ACE2^-/y^ (n = 6) and WT (n = 6) mice. Representative western blot and densitometric analysis of eNOS (**E**) and phosphorylated Ser1177-eNOS (**F**) protein levels in thoracic aortas from ACE2^-/y^ (n = 5) and WT (n = 5) mice. (**G**) Relative expression of phospho-eNOS normalized to the total eNOS levels. (**H**) eNOS mRNA expression levels (real time PCR analysis). Data are expressed as mean ± SEM. *P<0.05, **P<0.01, **P<0.001, Student’s *t* test.

We next tested if ^•^NO levels are affected by ACE2-deficiency. Determination of nitrite and nitrate (NOx), stable ^•^NO metabolites, in body fluids is widely used as a marker of ^•^NO production [29]. Both plasma nitrite concentration (Fig 2A) and the urinary NOx excretion rates (Fig 2B) were markedly decreased in ACE2^-/y^ mice in comparison to WT animals. These results indicate that ^•^NO production is impaired in ACE2-deficient mice.

eNOS is the main source of plasma ^•^NO. To evaluate eNOS activity, we next investigated ^•^NO release *in vitro* in aortic segments of ACE2^-/y^ mice. ACh-induced ^•^NO release was decreased in ACE2^-/y^ mice compared to WT animals (Fig 2C). This effect was blunted by the eNOS inhibitor L-NAME (Fig 2C). To exclude the possibility that the decrease in ^•^NO production was due to an altered activity of arginase, an enzyme which competes with NOS isoforms for the same substrate, L-arginine, we also measured activity of this enzyme. No significant difference was found in total arginase activity (Fig 2D) between ACE2^-/y^ and WT mice, arguing that decrease in the ^•^NO production was due to a reduction in eNOS activity.

**Fig 3:**
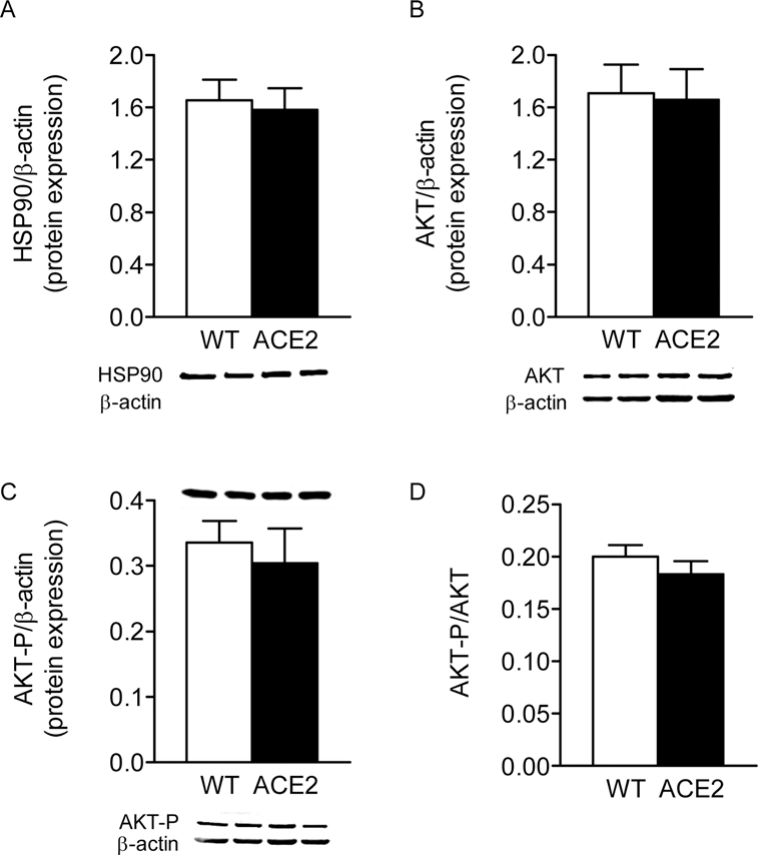
AKT expression in ACE2^-/y^ mice. Representative western blots and densitometric analysis of HSP90 (**A**), AKT (**B**) and P-AKT (**C**) aortic protein levels. (**D**) P-AKT/AKT ratio. Results are representative of four to six separate experiments. Data are expressed as mean ± SEM.

eNOS activity is regulated by phosphorylation at multiple sites. Therefore we evaluated the phosphorylation status of eNOS in ACE2^-/y^ mice. Both the amount of total eNOS protein and eNOS phosphorylated at the activation site Ser1177 (Fig 2E and 2F) were significantly decreased in aortic tissue of ACE2^-/y^ compared to WT mice, whereas the relation between P-eNOS Ser1177 and total eNOS was unaffected by ACE2-deficiency (Fig 2H). Moreover, eNOS mRNA expression was reduced in aorta of ACE2^-/y^ mice, further confirming a downregulation of eNOS expression evoked by the lack of ACE2. The HSP90 (Fig 3A), AKT (Fig 3B), P-AKT (Fig 3C), and P-AKT/AKT ratio (Fig 3D) remained unchanged between strains, suggesting that the impairment in ^•^NO generation observed in ACE2^-/y^ mice is driven by AKT-independent pathways.

Taken together, our findings showed that ACE2 deficiency results in the reduction of ^•^NO bioavailability due to the downregulation of the eNOS expression.

### ACE2-deficiency results in increased oxidative stress

**Fig 4:**
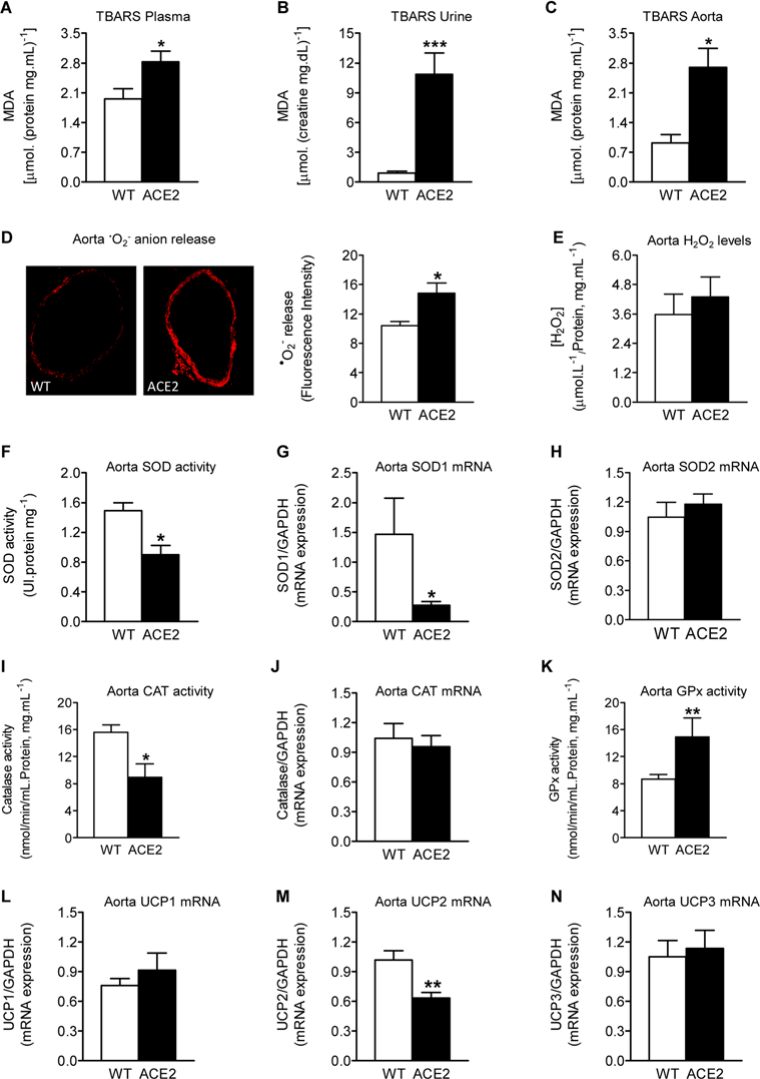
Oxidative stress and ROS degrading enzymes in ACE2^-/y^ mice. Concentration of the TBARS malondialdehyde (MDA) in plasma (**A**), urine (**B**), and aorta (**C**) of ACE2^-/y^ (n = 5) and WT (n = 5) mice. (**D**) Representative fluorescent photomicrographs (left) and quantification (right) showing *in situ* detection of ^⚫^O_2_^-^ by confocal microscopy (7 μm sections of thoracic aorta). Aortic sections were labeled with the oxidative dye dihydroethidium (2 μM/L), which is oxidized to EtBr in the presence of ^⚫^O_2_^-^ and gives red fluorescence. High EtBr fluorescence was found in aortic walls of ACE2^-/y^ mice, whereas the signal was almost undetectable in aortas of WT animals. (n = 5–6). (**E**) H_2_O_2_ levels in aorta of ACE2^-/y^ mice. Activity (**F, I**) and mRNA expression by real time PCR (**G, H, J**) of ROS degrading enzymes, SOD (**F, G, H**) and catalase (**I, J**) in aorta of ACE2^-/y^ (n = 4) and WT (n = 5) mice. (**K**) aorta GPx activity. mRNA expression level of UCP1 (**L**), UCP2 (**M**), and UCP3 (**N**) in aorta of ACE2^-/y^ mice. *P<0.05, **P<0.01 (Student *t* test).

^•^NO is a known antioxidant, which provides a protective function against ROS action by quenching ^•^O_2_^−^. We hypothesized that reduced ^•^NO availability may contribute to the increased oxidative stress in ACE2^-/y^ mice. Indeed, evaluation of lipid peroxidation, which is an indicator of oxidative stress, revealed a significant increase in the levels of the TBARS, malondialdehyde (MDA), in plasma, urine, and aorta of ACE2^-/y^ mice (Fig 4A–4C). Also direct measurement of the ^•^O_2_^-^ release in the aorta showed an elevation in oxidative stress in ACE2^-/y^ mice in comparison to WT animals (Fig 4D). However, ACE2 deletion did not affect aortic concentrations of H_2_O_2_ (Fig 4E), a ^•^O_2_^-^ dismutation product generated by superoxide dismutase (SOD). Analysis of enzymes involved in ROS metabolism showed a significant decrease in both total SOD and catalase activity in aorta of ACE2^-/y^ mice (Fig 4F and 4I, respectively). Concurrently, the mRNA levels of SOD1 (Fig 4G), but not SOD2 (Fig 4H) and catalase (Fig 4J) were lower in ACE2^-/y^ than in WT mice. Interestingly, under similar experimental conditions, the aortic glutathione peroxidase (GPx) activity in ACE2^–/y^ mice was ~2-fold higher than that of WT animals (Fig 4K).

Since enhanced oxidative stress was observed in aorta of ACE2^-/y^ mice, we analyzed the expression of uncoupling protein 2 (UCP2), an inner-mitochondrial key regulator of intracellular ROS, by real-time PCR analysis using total RNA from aorta tissue. ACE2 deletion induced a significant decrease in UCP2 mRNA levels (Fig 4M), compared with the control animals. In contrast, the expression of other isoforms of UCP, UCP1 and UCP3, were not changed (Fig 4L–4N).

Together, these results suggest that oxidative stress observed in ACE2^-/y^ mice is mainly caused by inefficient antioxidation and scavenging of ROS.

## Discussion

ACE2 is a monocarboxypeptidase, whose main function in the RAS is the degradation of the proinflammatory and vasoconstrictor peptide, Ang II, and the generation of Ang-(1–7), which is known to counterbalance the actions of Ang II [18]. We took advantage of a mouse model with genetic deletion of ACE2 to address the role of this enzyme in the regulation of the balance between ^•^O_2_^-^ and released ^•^NO within the endothelial environment which is pivotal in the maintenance of vascular homeostasis [18,30]. The major finding of the present study demonstrated the establishment of a novel equilibrium in the vascular wall of in ACE2-deficient mice through processes associated with increased aortic oxidative stress and decreased ^•^NO release.

In our study, we have shown that genetic ablation of ACE2 in mice on a C57BL/6 genetic background leads to a small but significant elevation in blood pressure, which is probably the result of vascular dysfunction. These data are consistent with a previous study showing increase in blood pressure in ACE2^-/y^ mice on C57BL/6 background [31], but does not concur with other studies in different mouse strains [4,31,32]. Other groups may have missed the small change in blood pressure due to the mixed genetic background, high variability of the blood pressure data and a lack of statistical power. The potential reasons for these discrepant results have been comprehensively discussed [33]. Interestingly, the backcross of the ACE2-null allele to the FVB/N background led to a more dramatic blood pressure upregulation in the resulting animals (our unpublished data), similar to the phenotype observed in Mas-deficient mice on the C57BL/6 [5] and on the FVB/N [19] backgrounds, suggesting a protective role of the C57BL/6 background in blood pressure regulation.

Interestingly, the vascular dilatation induced by bolus intra-aortic injections of increasing doses of ACh was not altered in ACE2-deficient mice, despite that a drastic reduction in ^•^NO bioavailability was observed. However, the response to the endothelium independent vasodilator, SNP, was enhanced in ACE2^-/y^ mice, pointing to an adaptation process happening under chronic removal of ACE2. These *in-vivo* observations were reproduced *in vitro* in isolated aortic rings and are, therefore, not caused by any systemic alterations in these mice. Probably, the altered SNP-response of the smooth muscle layer is induced by the chronic reduction in eNOS activity in ACE2-deficient animals, since a similar phenomenon was observed in eNOS-deficient mice: SNP induced a markedly greater accumulation of cGMP in aortic rings of these animals compared to controls [34]. The underlying molecular mechanisms, however, remained elusive but involved an increased activity of soluble guanylate cyclase.

In this study we observed a drastic reduction in ^•^NO availability in ACE2^-/y^ mice, which was obvious from a reduced ^•^NO release from intact aortic rings and a strong decrease in urinary concentration of NOx and plasmatic nitrite in ACE2-deficient mice. Nitrite serves as an indirect indicator of endothelial ^•^NO release, and is reduced also in humans with endothelial dysfunction [29]. According to our data, these reduced levels of ^•^NO in ACE2^-/y^ mice are due to two different effects of ACE2 depletion: a reduction in active eNOS and an inactivation of ^•^NO by ROS. On one hand, the amount of phosphorylated eNOS at Ser1177, which modulates both the calcium sensitivity and activity of the enzyme, was significantly reduced in ACE2^-/y^ mice compared to control animals. Since ACE2 deletion did not alter the AKT pathway, the decrease in eNOS activity must be regulated by AKT-independent mechanisms [35] and could at least partially be explained by its lowered expression level, whereby the mechanisms leading to this downregulation of eNOS expression need further elucidation. On the other hand, increased oxidative stress observed in ACE2-deficient mice may lead to the depletion of BH_4_ [36], a cofactor essential for the coupling of eNOS, and thereby shift the balance between ^•^NO and ^•^O_2_ generation by eNOS to the favor of the second [37,38], further decreasing ^•^NO levels and increasing ROS production in these mice. eNOS uncoupling is an important mechanism leading to pathologic ^•^O_2_^-^ release in the vascular endothelium, which induces decline in ^•^NO availability. Quick oxidative inactivation of ^•^NO by ^•^O_2_^-^, and consequent formation of ^-^ONOO, a potent oxidant [39], plays a pivotal role in the development of endothelial dysfunction [40]. These events are associated with an increase in lipid peroxidation, as we observed in the present study by monitoring increased systemic and aortic TBARS levels. Increased superoxide and peroxynitrite levels have also recently been found by other groups in ACE2^-/y^ mice at baseline or after Ang II infusion and have been linked to increased NADPH oxidase activity [41,42].

The increased ROS levels observed in ACE2^-/y^ mice can be also caused by a reduction in antioxidant capacity in these animals. This hypothesis is supported by a significant reduction in aortic SOD and catalase activity in ACE2^-/y^ mice, enzymes, which inactivate ^•^O_2_^-^ [39]. and convert H_2_O_2_ to molecular oxygen and water [43]., respectively. These results are in accordance with those previously demonstrating that exogenous SOD improves the endothelium-dependent vascular relaxation response to ACh at basal conditions [44]. Moreover, increasing vascular SOD activity induces a significant improvement in arterial relaxation in cholesterol-fed rabbits [45]. Unexpectedly, the levels of H_2_O_2_ in aorta were similar in both strains. This may perhaps be due to the increase of GPx and the decrease of catalase activities in the aortas of ACE^-/y^ mice, which counterbalance each other and normalize the H_2_O_2_ levels, since both enzymes are responsible for H_2_O_2_ degradation [43,46].

It has been shown that UCP2, an inner-mitochondrial key regulator of intracellular ROS, preserves endothelial function in obese diabetic mice [47] and prevents salt-sensitive hypertension [48] by promoting the increase in ^•^NO bioavailability and suppressing ROS production. In agreement with these previous findings, our data demonstrate that ACE2 null mice show a significant decrease in UCP2 mRNA levels in aorta which may directly contribute to the increase in ^•^O_2_^-^ and decreased ^•^NO bioavailability and, consequently, to endothelial dysfunction. Accordingly, UCP2 upregulation was recently observed in isolated endothelial cells overexpressing ACE2 [49].

Taken together, our current work provides strong evidence for a disturbance of the oxidative/nitrosative balance in ACE2-deficient mice. Concurrently, Bodiga and colleagues [14] showed an exaggerated increase in ^•^O_2_^-^ production, probably by NAD(P)H oxidase following pressure-overload induced by aortic constriction in ACE2^-/y^ mice. The authors showed that Ang-(1–7) delivery by mini-osmotic pumps decreases the NAD(P)H oxidase activity and improves the early dilated cardiomyopathy in pressure-overloaded animals. Jin et al. [39], showed that endothelial dysfunction induced by Ang II infusion is exaggerated in ACE2-deficient mice mainly by hyperactive ROS generation. In hypertensive rats, overexpressing human ACE2 in VSMC endothelium-dependent relaxation increased in response to ACh in both, *in vitro* and *in vivo* conditions [20]. Furthermore, we and others [49,50] recently showed that pharmacological activation of ACE2 ameliorates endothelial dysfunction in hypertensive and diabetic animals. In two different mouse models lacking Mas, a central component of ACE2/Ang/(1–7)/Mas axis, we have shown that the increase in local and systemic oxidative stress, observed in these mice, was strongly correlated with *in vivo* endothelial dysfunction [19]. Hence, our and other reports show that the ACE2/Ang-(1–7)/Mas arm of the RAS regulates endothelial function by modulating the oxidative stress and ^•^NO release balance.

Several studies have previously shown that ^•^NO is a potent and central mediator of several physiopathological processes involved in cardiovascular regulation [18,39,51,52]. Moreover, a number of studies in patients with cardiovascular diseases have described a close relationship between imbalance of vascular ^•^NO and ROS concentrations and endothelial dysfunction [52,53]. Hypertensive patients have been reported to have significantly higher levels of plasma lipid peroxidation when compared with normotensive subjects [54]. Accordingly, plasma MDA, an index of lipid peroxidation, was significantly and positively correlated with endothelial dysfunction in both humans [51,55] and animal models [19,56]. Thus, it is conceivable that in hypertension and other cardiovascular diseases an imbalance between the protective ACE2/Ang-(1–7)/Mas arm, and the deleterious ACE/AngII/AT1 axis of the RAS induces a significant reduction in ^•^NO bioavailability associated with oxidative stress and causes endothelial dysfunction.

In conclusion, we elucidate mechanisms by which ACE2 is involved in the maintenance of vascular homeostasis. Furthermore, these findings provide insights into the role of the RAS in both vascular and systemic redox balance. Therefore, the ACE2/Ang-(1–7)/Mas axis is a potential target for the development of novel cardiovascular protective and/or antioxidant drugs.

## Supporting Information

S1 Fig Heart rate before and after treatment of ACE2^-/y^ and WT mice with ACh [50–200 μg.kg^-1^] and 10 μg.kg^-1^ SNP by injection in the descending aorta. There is no direct cardiodepressive effect by ACh on the heart but even at some doses a slight increase in heart rate after injection probably induced by the baroreflex. *P<0.05 (paired *t* test).
(PDF)

## References

[1] Bauer UE, Briss PA, Goodman RA, Bowman BA. (2014) Prevention of chronic disease in the 21st century: elimination of the leading preventable causes of premature death and disability in the USA. Lancet 384: 45–52. 10.1016/S0140-6736(14)60648-6 24996589

[2] Cosentino F, Barker JE, Brand MP, Heales SJ, Werner ER, Tippins JR, et al (2001) Reactive oxygen species mediate endothelium-dependent relaxations in tetrahydrobiopterin-deficient mice. Arterioscler Thromb Vasc Biol 21: 496–502. 11304463 10.1161/01.atv.21.4.496

[3] Shi Y, Vanhoutte PM. (2009) Reactive oxygen-derived free radicals are key to the endothelial dysfunction of diabetes. J Diabetes 1: 151–162. 10.1111/j.1753-0407.2009.00030.x 20923534

[4] Crackower MA, Sarao R, Oudit GY, Yagil C, Kozieradzki I, Scanga SE, et al (2002) Angiotensin-converting enzyme 2 is an essential regulator of heart function. Nature 417: 822–828. 12075344 10.1038/nature00786

[5] Rabelo LA, Xu P, Todiras M, Sampaio WO, Buttgereit J, Bader M, et al (2008) Ablation of angiotensin (1–7) receptor Mas in C57Bl/6 mice causes endothelial dysfunction. J Am Soc Hypertens 2: 418–424. 10.1016/j.jash.2008.05.003 20409925

[6] Santos RA, Simoes e Silva AC, Maric C, Silva DM, Machado RP, de Buhr I, et al (2003) Angiotensin-(1–7) is an endogenous ligand for the G protein-coupled receptor Mas. Proc Natl Acad Sci USA 100: 8258–8263. 12829792 10.1073/pnas.1432869100 PMC166216

[7] Lovren F, Pan Y, Quan A, Teoh H, Wang G, Shukla PC, et al (2008) Angiotensin converting enzyme-2 confers endothelial protection and attenuates atherosclerosis. Am J Physiol 295: H1377–1384. 10.1152/ajpheart.00331.2008 18660448

[8] Thatcher SE, Zhang X, Howatt DA, Lu H, Gurley SB, Daugherty A, et al (2011) Angiotensin-converting enzyme 2 deficiency in whole body or bone marrow-derived cells increases atherosclerosis in low-density lipoprotein receptor-/- mice. Arterioscler Thromb Vasc Biol 31: 758–765. 10.1161/ATVBAHA.110.221614 21252069 PMC3086633

[9] Thomas MC, Pickering RJ, Tsorotes D, Koitka A, Sheehy K, Bernardi S, et al (2010) Genetic Ace2 deficiency accentuates vascular inflammation and atherosclerosis in the ApoE knockout mouse. Circ Res 107: 888–897. 10.1161/CIRCRESAHA.110.219279 20671240

[10] Shiota A, Yamamoto K, Ohishi M, Tatara Y, Ohnishi M, Maekawa Y, et al (2010) Loss of ACE2 accelerates time-dependent glomerular and tubulointerstitial damage in streptozotocin-induced diabetic mice. Hypertens Res 33: 298–307. 10.1038/hr.2009.231 20186149

[11] Wong DW, Oudit GY, Reich H, Kassiri Z, Zhou J, et al (2007) Loss of angiotensin-converting enzyme-2 (Ace2) accelerates diabetic kidney injury. Am J Pathol 171: 438–451. 17600118 10.2353/ajpath.2007.060977 PMC1934545

[12] Oudit GY, Kassiri Z, Jiang C, Liu PP, Poutanen SM, Penninger JM, et al (2009) SARS-coronavirus modulation of myocardial ACE2 expression and inflammation in patients with SARS. Eur J Clin Invest 39: 618–625. 10.1111/j.1365-2362.2009.02153.x 19453650 PMC7163766

[13] Alghamri MS, Weir NM, Anstadt MP, Elased KM, Gurley SB, Morris M. (2013) Enhanced angiotensin II-induced cardiac and aortic remodeling in ACE2 knockout mice. J Cardiovasc Pharmacol Ther 18: 138–151. 10.1177/1074248412460124 23043153

[14] Bodiga S, Zhong JC, Wang W, Basu R, Lo J, Liu GC, et al (2011) Enhanced susceptibility to biomechanical stress in ACE2 null mice is prevented by loss of the p47phox NADPH oxidase subunit. Cardiovasc Res 91: 151–161. 10.1093/cvr/cvr036 21285291 PMC3151662

[15] Moritani T, Iwai M, Kanno H, Nakaoka H, Iwanami J, Higaki T, et al (2013) ACE2 deficiency induced perivascular fibrosis and cardiac hypertrophy during postnatal development in mice. J Am Soc Hypertens 7: 259–266. 10.1016/j.jash.2013.03.002 23608725

[16] Yamamoto K, Ohishi M, Katsuya T, Ito N, Ikushima M, Kaibe M, et al (2006) Deletion of angiotensin-converting enzyme 2 accelerates pressure overload-induced cardiac dysfunction by increasing local angiotensin II. Hypertension 47: 718–726. 16505206 10.1161/01.HYP.0000205833.89478.5b

[17] Shi Y, So KF, Man RY, Vanhoutte PM. (2007) Oxygen-derived free radicals mediate endothelium-dependent contractions in femoral arteries of rats with streptozotocin-induced diabetes. Br J Pharmacol 152: 1033–1041. 17767168 10.1038/sj.bjp.0707439 PMC2095103

[18] Rabelo LA, Alenina N, Bader M. (2011) ACE2-angiotensin-(1–7)-Mas axis and oxidative stress in cardiovascular disease. Hypertens Res 34: 154–160. 10.1038/hr.2010.235 21124322

[19] Xu P, Costa-Goncalves AC, Todiras M, Rabelo LA, Sampaio WO, Moura MM, et al (2008) Endothelial dysfunction and elevated blood pressure in MAS gene-deleted mice. Hypertension 51: 574–580. 10.1161/HYPERTENSIONAHA.107.102764 18180400

[20] Rentzsch B, Todiras M, Iliescu R, Popova E, Campos LA, Oliveira ML, et al (2008) Transgenic angiotensin-converting enzyme 2 overexpression in vessels of SHRSP rats reduces blood pressure and improves endothelial function. Hypertension 52: 967–973. 10.1161/HYPERTENSIONAHA.108.114322 18809792

[21] Patel SK, Wai B, Ord M, MacIsaac RJ, Grant S, Velkoska E, et al (2012) Association of ACE2 genetic variants with blood pressure, left ventricular mass, and cardiac function in Caucasians with type 2 diabetes. Am J Hypertens 25: 216–222. 10.1038/ajh.2011.188 21993363

[22] Pena Silva RA, Chu Y, Miller JD, Mitchell IJ, Penninger JM, Faraci FM, et al (2012) Impact of ACE2 deficiency and oxidative stress on cerebrovascular function with aging. Stroke 43: 3358–3363. 10.1161/STROKEAHA.112.667063 23160880 PMC3529166

[23] Fesus G, Dubrovska G, Gorzelniak K, Kluge R, Huang Y, Luft FC, et al (2007) Adiponectin is a novel humoral vasodilator. Cardiovasc Res. 75: 719–727 17617391 10.1016/j.cardiores.2007.05.025

[24] Bradford MM. (1976) A rapid and sensitive method for the quantitation of microgram quantities of protein utilizing the principle of protein-dye binding. Anal Biochem 72: 248–254. 942051 10.1016/0003-2697(76)90527-3

[25] White AR, Ryoo S, Li D, Champion HC, Steppan J, Wang D, et al (2006) Knockdown of arginase I restores NO signaling in the vasculature of old rats. Hypertension 47: 245–251. 16380531 10.1161/01.HYP.0000198543.34502.d7

[26] Peshavariya HM, Dusting GJ, Selemidis S. (2007) Analysis of dihydroethidium fluorescence for the detection of intracellular and extracellular superoxide produced by NADPH oxidase. Free Radic Res 41: 699–712. 17516243 10.1080/10715760701297354

[27] Vinh A, Widdop RE, Drummond GR, Gaspari TA. (2008) Chronic angiotensin IV treatment reverses endothelial dysfunction in ApoE-deficient mice. Cardiovasc Res 77: 178–187. 18006471 10.1093/cvr/cvm021

[28] Ohkawa H, Ohishi N, Yagi K. (1979) Assay for lipid peroxides in animal tissues by thiobarbituric acid reaction. Anal Biochem 95: 351–358. 36810 10.1016/0003-2697(79)90738-3

[29] Kleinbongard P, Dejam A, Lauer T, Jax T, Kerber S, Gharini P, et al (2006) Plasma nitrite concentrations reflect the degree of endothelial dysfunction in humans. Free Radic Biol Med 40: 295–302. 16413411 10.1016/j.freeradbiomed.2005.08.025

[30] Mueller CF, Laude K, McNally JS, Harrison DG. (2005) ATVB in focus: redox mechanisms in blood vessels. Arterioscler Thromb Vasc Biol 25: 274–278. 15514203 10.1161/01.ATV.0000149143.04821.eb

[31] Gurley SB, Allred A, Le TH, Griffiths R, Mao L, Philip N, et al (2006) Altered blood pressure responses and normal cardiac phenotype in Ace2-null mice. J Clin Invest 116: 2218–2225 16878172 10.1172/JCI16980 PMC1518789

[32] Moritani T, Iwai M, Kanno H, Nakaoka H, Iwanami J, Higaki T, et al (2013) Ace2 deficiency induced perivascular fibrosis and cardiac hypertrophy during postnatal development in mice. J Am Soc Hypertens 7: 259–266 10.1016/j.jash.2013.03.002 23608725

[33] Gurley SB, Coffman TM. (2008) Angiotensin-converting enzyme 2 gene targeting studies in mice: mixed messages. Exp Physiol 93: 538–542 10.1113/expphysiol.2007.040014 18376006

[34] Brandes RP, Kim D, Schmitz-Winnenthal FH, Amidi M, Godecke A, Mulsch A, et al (2000) Increased nitrovasodilator sensitivity in endothelial nitric oxide synthase knockout mice: role of soluble guanylyl cyclase. Hypertension 35: 231–236. 10642303 10.1161/01.hyp.35.1.231

[35] Rafikov R, Fonseca FV, Kumar S, Pardo D, Darragh C, Elms S, et al (2011) eNOS activation and NO function: structural motifs responsible for the posttranslational control of endothelial nitric oxide synthase activity. J Endocrinol 210: 271–284. 10.1530/JOE-11-0083 21642378 PMC3326601

[36] Dumitrescu C, Biondi R, Xia Y, Cardounel AJ, Druhan LJ, Ambrosio G, et al (2007) Myocardial ischemia results in tetrahydrobiopterin (BH4) oxidation with impaired endothelial function ameliorated by BH4. Proc Natl Acad Sci USA 104: 15081–15086. 17848522 10.1073/pnas.0702986104 PMC1986616

[37] Chen CA, Druhan LJ, Varadharaj S, Chen YR, Zweier JL. (2008) Phosphorylation of endothelial nitric-oxide synthase regulates superoxide generation from the enzyme. J Biol Chem 283: 27038–27047. 10.1074/jbc.M802269200 18622039 PMC2556006

[38] Vasquez-Vivar J, Kalyanaraman B, Martasek P, Hogg N, Masters BS, Karoui H, et al (1998) Superoxide generation by endothelial nitric oxide synthase: the influence of cofactors. Proc Natl Acad Sci USA 95: 9220–9225. 9689061 10.1073/pnas.95.16.9220 PMC21319

[39] Pacher P, Beckman JS, Liaudet L. (2007) Nitric oxide and peroxynitrite in health and disease. Physiol Rev 87: 315–424. 17237348 10.1152/physrev.00029.2006 PMC2248324

[40] Berka V, Wu G, Yeh HC, Palmer G, Tsai AL. (2004) Three different oxygen-induced radical species in endothelial nitric-oxide synthase oxygenase domain under regulation by L-arginine and tetrahydrobiopterin. J Biol Chem 279: 32243–32251. 15166218 10.1074/jbc.M404044200

[41] Jin HY, Song B, Oudit GY, Davidge ST, Yu HM, Jiang YY, et al (2012) ACE2 deficiency enhances angiotensin II-mediated aortic profilin-1 expression, inflammation and peroxynitrite production. PLoS One 7: e38502 10.1371/journal.pone.0038502 22693641 PMC3367919

[42] Patel VB, Zhong JC, Fan D, Basu R, Morton JS, Parajuli N, et al (2014) Angiotensin-converting enzyme 2 is a critical determinant of angiotensin II-induced loss of vascular smooth muscle cells and adverse vascular remodeling. Hypertension 64: 157–164. 10.1161/HYPERTENSIONAHA.114.03388 24799609

[43] Deisseroth A, Dounce AL. (1970) Catalase: Physical and chemical properties, mechanism of catalysis, and physiological role. Physiol Rev 50: 319–375. 4912904 10.1152/physrev.1970.50.3.319

[44] Miller FJ Jr, Gutterman DD, Rios CD, Heistad DD, Davidson BL. (1998) Superoxide production in vascular smooth muscle contributes to oxidative stress and impaired relaxation in atherosclerosis. Circ Res 82: 1298–1305. 9648726 10.1161/01.res.82.12.1298

[45] Mugge A, Elwell JH, Peterson TE, Hofmeyer TG, Heistad DD, Harrison DG. (1991) Chronic treatment with polyethylene-glycolated superoxide dismutase partially restores endothelium-dependent vascular relaxations in cholesterol-fed rabbits. Circ Res 69: 1293–1300. 1934359 10.1161/01.res.69.5.1293

[46] Lei XG, Cheng WH, McClung JP. (2007) Metabolic regulation and function of glutathione peroxidase-1. Annu Rev Nutr 27: 41–61. 17465855 10.1146/annurev.nutr.27.061406.093716

[47] Tian XY, Wong WT, Xu A, Lu Y, Zhang Y, Wang L, et al (2012) Uncoupling protein-2 protects endothelial function in diet-induced obese mice. Circ Res 110: 1211–1216. 10.1161/CIRCRESAHA.111.262170 22461387

[48] Botelho-Santos GA, Bader M, Alenina N, Santos RA. (2012) Altered regional blood flow distribution in Mas-deficient mice. Ther Adv Cardiovasc Dis 6: 201–211. 10.1177/1753944712461164 23045193

[49] Zhang Y, Liu J, Luo JY, Tian XY, Cheang WS, Xu J, et al (2015) Upregulation of Angiotensin (1–7)-Mediated Signaling Preserves Endothelial Function Through Reducing Oxidative Stress in Diabetes. Antioxid Redox Signal. 23: 880–892. 10.1089/ars.2014.6070 25867182 PMC4617412

[50] Fraga-Silva RA, Costa-Fraga FP, Murca TM, Moraes PL, Martins Lima A, Lautner RQ, et al (2013) Angiotensin-converting enzyme 2 activation improves endothelial function. Hypertension 61: 1233–1238. 10.1161/HYPERTENSIONAHA.111.00627 23608648 PMC3733257

[51] Higashi Y, Sasaki S, Nakagawa K, Matsuura H, Oshima T, Chayama K. (2002) Endothelial function and oxidative stress in renovascular hypertension. N Engl J Med 346: 1954–1962. 12075056 10.1056/NEJMoa013591

[52] Touyz RM, Briones AM. (2011) Reactive oxygen species and vascular biology: implications in human hypertension. Hypertens Res 34: 5–14. 10.1038/hr.2010.201 20981034

[53] Wilkinson IB, MacCallum H, Cockcroft JR, Webb DJ. (2002) Inhibition of basal nitric oxide synthesis increases aortic augmentation index and pulse wave velocity in vivo. Br J Clin Pharmacol 53: 189–192. 11851643 10.1046/j.1365-2125.2002.1528adoc.x PMC1874288

[54] Bandeira Sde M, Guedes Gda S, da Fonseca LJ, Pires AS, Gelain DP, Moreira JC, et al (2012) Characterization of blood oxidative stress in type 2 diabetes mellitus patients: increase in lipid peroxidation and SOD activity. Oxid Med Cell Longev 2012: 819310 10.1155/2012/819310 23259029 PMC3509371

[55] Saraswathi R, Sankar D, Ali A, Uehara Y, Abe S, Sambandam G, et al (2011) A Pilot Assessment of Oxidative Stress Byproducts and Antioxidant Activities Among Indian Patients with Various Stages of Hypertension. Clin Exp Hypertens. 33:437–443 10.3109/10641963.2010.549259 21627488

[56] Briones AM, Rodriguez-Criado N, Hernanz R, Garcia-Redondo AB, Rodrigues-Diez RR, Alonso MJ, et al (2009) Atorvastatin prevents angiotensin II-induced vascular remodeling and oxidative stress. Hypertension 54: 142–149. 10.1161/HYPERTENSIONAHA.109.133710 19451411

