# Supplementary material for: Genetic Deletion of ACE2 Induces Vascular Dysfunction in C57BL/6 Mice: Role of Nitric Oxide Imbalance and Oxidative Stress
Source: PLoS One. 2016 Apr 12;11(4):e0150255. doi: 10.1371/journal.pone.0150255 (PMC4829150; doi:10.1371/journal.pone.0150255)
Supplement: S1 Fig — There is no direct cardiodepressive effect by ACh on the heart but even at some doses a slight increase in heart rate after injection probably induced by the baroreflex. *P<0.05 (paired t test). (PDF) [file pone.0150255.s001.pdf]

Supporting Information

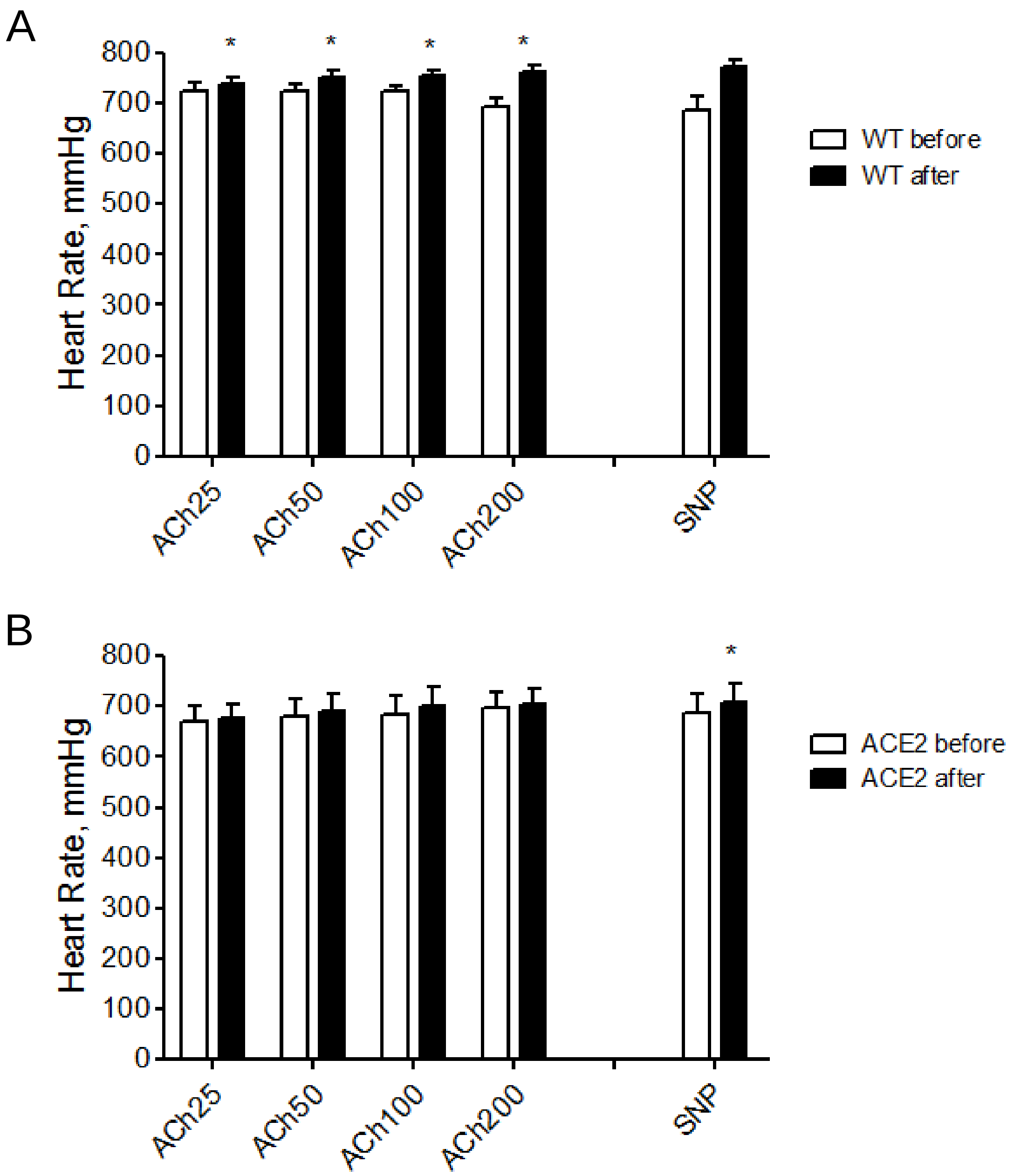

S1 Fig. Heart rate before and after treatment of ACE2$^{-/y}$ and WT mice with Ach (25-200 ng/kg) and SNP (10 µg/kg) by injection in the descending aorta. There is no direct cardiodepressive effect by ACh on the heart but even at some doses a slight increase in heart rate after injection probably induced by the baroreflex. *P<0.05 (paired t test)**.**
